# Effect of Chlorogenic Acids on Cognitive Function: A Randomized, Double-Blind, Placebo-Controlled Trial

**DOI:** 10.3390/nu10101337

**Published:** 2018-09-20

**Authors:** Katsuyoshi Saitou, Ryuji Ochiai, Kazuya Kozuma, Hirotaka Sato, Takashi Koikeda, Noriko Osaki, Yoshihisa Katsuragi

**Affiliations:** 1 Health Care Food Research Laboratories, Kao Corporation, 2-1-3 Bunka, Sumida-ku, Tokyo 131-8501, Japan; saitou.katsuyoshi@kao.com (K.S.); kouzuma.kazuya@kao.com (K.K.); katsuragi.yoshihisa@kao.com (Y.K.); 2 Biological Science Laboratories, Kao Corporation, 2-1-3 Bunka, Sumida-ku, Tokyo 131-8501, Japan; osaki.noriko@kao.com; 3 Development Research-Health Care/Household, Kao Corporation, 2-1-3 Bunka, Sumida-ku, Tokyo 131-8501, Japan; sato.hirotaka@kao.com; 4 Shiba Pales Clinic, 1-9-10 Hamamatsucho, Minato-ku, Tokyo 105-0013, Japan; jimukyoku@mail.souken-r.com

**Keywords:** chlorogenic acid, cognitive functions, psychomotor speed

## Abstract

(1) Background: Chlorogenic acids (CGAs) have been attracting interest of late, owing to their health benefits. Here, we performed a randomized, double-blind, placebo-controlled trial to investigate whether CGAs improved cognitive function in humans. (2) Methods: Thirty-eight healthy participants were assigned to either the CGA group, which was given CGA-added beverage daily for 16 weeks, or the placebo group. Cognitive functions were assessed using the Japanese version of the CNS Vital Signs (Cognitrax). (3) Results: The CGA group showed significant increase in the Cognitrax domain scores for motor speed, psychomotor speed, and executive function compared with the placebo group, as well as an improvement in the shifting attention test scores. In blood analysis, the CGA group showed increased levels of apolipoprotein A1 and transthyretin, both of which are putative biomarkers for early-stage cognitive decline. (4) Conclusions: These results suggest that CGAs may improve some cognitive functions, which would help in the efficient performance of complex tasks.

## 1. Introduction

Cognitive function encompasses a wide range of brain functions, such as memory, attention, language, and executive function, all of which are essential for our daily activities. Cognitive decline threatens independence and quality of life during old age [1,2]. Cross-sectional studies have shown that cognitive function starts declining gradually in the late 20s, and the decline is accelerated by neurodegenerative diseases, including Alzheimer’s disease (AD) [3]. Therefore, early intervention for maintaining normal cognitive function is one of the important factors for successful aging [4].

Epidemiological studies indicate that the progression of cognitive decline is considerably influenced by various lifestyle factors, including diet [5,6,7,8,9]. For example, long-term intake of the Mediterranean diet is associated with better cognition in older adults [9]. In recent years, polyphenols have gained considerable attention owing to their potential in preserving cognitive function and preventing neurodegenerative disorders [8,10,11,12,13]. A 3-year follow-up study found that higher levels of total urinary polyphenols, which are objective biomarkers of total dietary polyphenol intake, were indicative of a lower risk of cognitive decline [10]. Another cross-sectional study showed that consumption of flavonoid-rich foods, including chocolate, wine, and tea, was associated with better performance in cognitive tests in the elderly [11]. Furthermore, an inverse association between dietary polyphenol intake and the incidence of dementia has also been reported [12]. Yet, the mechanism underlying the effects of polyphenols on cognitive function remains unclear. A wide range of attributes of polyphenols are thought to be involved with neuronal preservation, including those that protect cells from oxidative stress, stimulate neurogenesis, increase cerebral blood flow, and improve metabolic disorders [8,12,13].

Chlorogenic acids (CGAs) are polyphenols abundant in coffee, which have been shown to be promising in preventing neurodegenerative disorders [14]. Regular coffee consumption reduces the risk of neurodegenerative conditions [15,16], and CGAs are believed to be the key contributors [17]. This is supported by the fact that CGAs and their metabolites confer neuroprotective effects against oxidative stress [18,19] and promote neuronal differentiation [20]. A number of rodent-model studies have also demonstrated that CGAs can improve learning and memory functions [14,17,21,22]. Moreover, CGAs have been shown to ameliorate vascular function and glucose tolerance [17].

While many studies have shown positive effects of CGAs on brain function, very few human clinical studies have been conducted so far. Cropley et al. showed that the acute administration of CGAs exerts some positive mood and mood-related behavioral effects; however, they did not find any cognitive-improving effects [23], with similar results reported by Camfield et al. [24]. Recently, we performed a preliminary open-label trial to elucidate whether CGAs were beneficial for improving cognitive function in the elderly. In the trial, a 6-month intake of CGAs led to improved scores on cognitive test batteries, suggesting that CGAs conferred cognitive-improving benefits [25]. To further demonstrate the beneficial potential of CGAs, we performed a randomized, double-blind, placebo-controlled trial involving healthy participants.

## 2. Materials and Methods 

### 2.1. Participants

Healthy voluntary participants aged 50–69 years with subjective memory complaints were recruited from around the Tokyo metropolitan area. The participants were preliminarily assessed for eligibility using questionnaires. After prescreening, the selected participants underwent a medical interview and two neuropsychological tests: a Mini-Mental State Examination (MMSE), a brief 30-point questionnaire widely used in screening for dementia [26], and the Japanese version of the Repeatable Battery for the Assessment of Neuropsychological Status (RBANS), a brief test comprising 12 subtests that measure various neurocognitive domains [27]. To exclude those with likely dementia, the selection criterion for MMSE scores was 24 or over. The participants with an MMSE score of 29 or 30 were also excluded, since their cognitive functions are considered completely normal. Other exclusion criteria were as follows: food allergies; history of surgery for cerebrovascular diseases; eating disorders; excessive smoking; heavy drinking; severe anemia; history of medication for hepatitis; epileptic seizure; diabetes; thyroid or renal dysfunction; previous experience taking neuropsychological tests in a hospital; having taken RBANS within the previous 3 months; participating in another clinical trial; having taken medicine or supplements that might influence cognitive function; drinking more than 1 cup of coffee per day; and those who were judged inappropriate for participation by the physician in charge. Written informed consent was obtained from all participants before the commencement of the study.

### 2.2. Experimental Design

The present study was designed as a randomized, double-blind, placebo-controlled, parallel-group trial with a 16-week experimental period. The participants were randomly allocated to a CGA group and a placebo group using a stratified randomization procedure based on MMSE and RBANS scores at screening. During the study period, the participants ingested either a CGA-added beverage or a placebo beverage daily 30–60 min before bedtime, since CGAs have sleep improving effects [28].

On the visit day, the participants received cognitive assessment and blood analysis. They were restricted from eating and drinking anything other than water, from 5 h before visiting the clinic until the end of all the tests. There were no other dietary restrictions, and the participants were instructed to maintain their usual lifestyle and dietary habits during the study period.

This study was performed at the Shiba Palace Clinic (Tokyo, Japan) from 3 December 2015 to 1 September 2016, in accordance with the Declaration of Helsinki (2013), managed by a contract research organization, SOUKEN CO., Ltd. (Tokyo, Japan). All the protocols were approved on 8 October 2015 by the Shiba Palace Clinic Ethics Committee, and were registered with the UMIN Clinical Trial Registry before the enrolment of the first participant (UMIN000020181). 

### 2.3. Materials

CGAs were extracted from green coffee beans by using a hot-water extraction method [29]. The extract was filtered to reduce caffeine levels to below the limit of quantification (<1 mg/100 g) to avoid the potential effects of caffeine on cognitive function and sleep quality [30,31]. The filtered extract was then spray-dried to obtain a dry powder. The CGAs in the extract comprised three types of compounds: caffeoylquinic acids (CQAs), feruloylquinic acids (FQAs), and dicaffeoylquinic acids (diCQAs). The composition of these CGA types, as assessed using high-performance liquid chromatography, was as follows: 67.5% CQAs, 13.8% FQAs, and 18.6% diCQAs. 

The CGA-added beverage was prepared using the coffee bean extract, water, acidifiers, amino acids, vitamins, sweeteners, and flavoring agents. The total amount of CQAs and FQAs was 300 mg. The placebo beverage was identical except that it contained no CGAs. 

### 2.4. Cognitive Function Assessment

For evaluating the cognitive function, the Japanese version of the CNS Vital Signs (CNSVS), known as Cognitrax, was performed at the baseline (0 weeks) and at 8 and 16 weeks after CGA or placebo treatment. CNSVS (CNS Vital Signs, LLC., Morrisville, NC, USA) is a computerized neurocognitive test battery that has been used extensively for its reliability and validity [32,33]. The test battery includes seven tests that are widely used in neuropsychological assessments: verbal memory test (VBM), visual memory test (VIM), finger tapping test (FTT), symbol digit cording (SDC), Stroop test (ST), shifting attention test (SAT), and continuous performance test (CPT). Tests were administered in this order.

In the two memory tests, the participants remember 15 words (VBM) or geometric figures (VIM). For immediate recognition, the participants identify those 15 targets nested in the 15 new non-targets (immediate recall). At the end of the battery, the 15 targets are presented again, mixed randomly among 15 new non-targets (delayed recall). The outcomes of these tests are the numbers of correct hits and passes.

In the FTT, the participants press the space bar on the keyboard with their right index finger as many times as they can in 10 s. This is repeated three times; then, the same trials are conducted with the left index finger. The outcomes are the average number of taps of the right or left hand. In the SDC, the references of eight symbol–digit pairs are presented on the screen. The participants type in as many of the number that corresponded to the highlighted symbol as possible in 120 s. The outcomes are the numbers of correct responses and errors.

The ST has three parts. In the first part, the black Japanese Kanji characters meaning red, yellow, blue, or green, appear randomly on the screen, and the participants respond as soon as they see any characters (simple reaction). In the second part, the differently colored Kanji characters appear on the screen, and the participants respond only when the color matches the meaning of the characters (complex reaction). In the third part, the differently colored Kanji characters appear on the screen, but the participants only respond when the color of the character does not match its meaning (Stroop reaction). The outcomes are reaction times of each part (simple reaction time, complex reaction time and Stroop reaction time) and the number of committed errors in the Stroop reaction test (Stroop commission errors). 

In the SAT, three figures appear on the screen, one on top and two at the bottom. The participants select one figure from the bottom that matches the top figure either by shape or by color according to the rule, which changes at random. The participants make as many correct matches as possible in 90 s. The outcomes are the number of correct responses and errors, and the reaction times of the correct responses.

In the CPT, English letters appear on the screen one by one, and the participants respond to “B” but not to any other letters for 5 min. In total, 200 letters are presented at random in the test, and 40 of these are the target letter “B”. The outcomes are the number of correct responses and errors, and reaction time.

As Table 1 shows, the scores obtained by performing the seven tasks generated a composite neurocognitive index (NCI) and 11 domain scores: composite memory, verbal memory, visual memory, psychomotor speed, reaction time, complex attention, cognitive flexibility, processing speed, executive function, and simple attention. For example, composite memory is calculated on the basis of the total number of correct hits and passes in the VBM and VIM. Detailed calculation methods for other domain scores are described elsewhere [32,33]. All individual test scores and domain scores were age-adjusted and standardized by setting the mean score as 100 and the standard deviation (SD) as 15, in which higher scores were always better. Standardized scores less than 20 were truncated at 20, as described previously [34].

### 2.5. Blood Analysis

Blood samples were collected at the baseline and at 16 weeks of CGA or placebo treatment. Serum concentrations of apolipoprotein A1 (ApoA1) and transthyretin (TTR) were measured at BML, Inc. (Tokyo, Japan). ApoA1 was evaluated by turbidimetric immunoassay with a commercially available kit (ApoA-I Auto-N’ Daiichi’, Sekisui Medical Co., Ltd., Tokyo, Japan). TTR was measured with a nephelometry kit (N Antiserum to human prealbumin, Siemens Healthcare Diagnostics Inc., Marburg, Germany). 

### 2.6. Statistical Analysis 

Data are presented as mean ± SD unless otherwise indicated. The baseline characteristics of the participants were analyzed using an unpaired *t*-test. The Cognitrax scores were analyzed by a two-way repeated measure ANCOVA with two time points (8 W–16 W) and groups (placebo and CGA) including the baseline score as covariance, which was followed by an unpaired *t*-test at each time point when the interaction was significant. The interaction between time and group, with three time points (0 W–16 W), was also analyzed using a liner mixed model (LMM). For the blood parameters, changes in the values of 0 W and 16 W were calculated (∆ values), and these were analyzed by an unpaired *t*-test. Statistical significance was set at *p* < 0.05, and two-sided. SPSS version 19 (IBM Inc, Tokyo, Japan) was used for all the statistical analysis. 

## 3. Results

### 3.1. Participants and Baseline Characteristics

**Figure 1:**
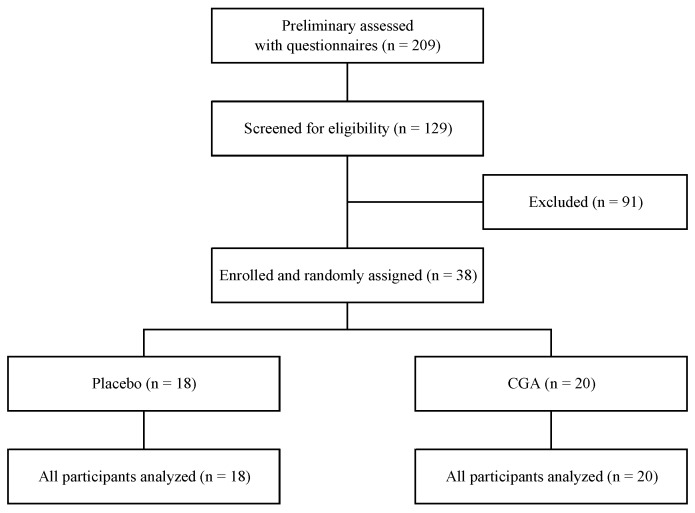
Flow diagram of the trial.

Recruitment of participants for this study was carried out in two phases. In total, 209 individuals were screened (144 during the first phase and 65 during the second) and, finally, 38 participants were enrolled. One participant whose MMSE score was 23 was included because the physician in charge judged that he did not have dementia. A participant flow chart is shown in Figure 1. All participants completed the trial by complying with the study protocol, which required the consumption of more than 80% of the test beverage. 

Baseline characteristics of the participants are presented in Table 2. None of the parameters measured at the baseline significantly differed between the placebo (*n* = 18) and CGA groups (*n* = 20). Throughout the study period, no adverse events were observed in relation to the intake of the test beverage.

### 3.2. Cognitive Functions

Table 3 shows the NCI and cognitive domain scores calculated in Cognitrax. In the ANCOVA analysis, a significant time × group interaction was observed in the scores of psychomotor speed, motor speed, and executive function. In addition, a marginal interaction was observed in the scores of cognitive flexibility. Pairwise comparison (unpaired *t*-test) revealed that the scores of psychomotor speed and executive function at 16 W in the CGA group tended to be higher than those in the placebo group (*p* = 0.066 and 0.061, respectively). For the main effect of time, significant changes were observed in NCI, verbal memory, complex attention, cognitive flexibility, and executive function.

In the LMM analysis (0 W–16 W), a significant time × group interaction was also obtained in the scores of psychomotor speed and motor speed.

Table 4 shows the scores of individual tests from Cognitrax. In the ANCOVA analyses, a significant time × group interaction was observed in the scores for right taps average in the FTT and correct responses in the SAT. In addition, a marginal interaction was observed in the scores of correct hits in the VBM, errors in the SDC, Stroop commission errors in the ST, and errors in the ST. Pairwise comparison showed the score for right taps average in the FTT, and correct responses in the SAT at 16 W in the CGA group tended to be higher than those in the placebo group (*p* = 0.071 and 0.063, respectively). For the main effect of group, in the CGA group, the scores for errors in the SDC tended to be higher (*p* = 0.080), and the scores for correct passes in the immediate VBM tended to be lower than those in the placebo group (*p* = 0.093), For the main effect of time, significant changes were observed in the scores for errors in the SDC, correct responses and errors in the SAT, and reaction time in the CPT.

In the LMM analysis (0 W–16 W), a significant time × group interaction was also observed in the scores for right taps average in the FTT.

### 3.3. Blood Analysis

Table 5 shows the results of blood analysis. With respect to ApoA1 concentration, values in the CGA group tended to be higher than that of the placebo group (*p* = 0.070), whereas values of TTR concentration were significantly higher of the CGA group than those of the placebo group. 

## 4. Discussion

To the best of our knowledge, this is the first randomized, double-blind, placebo-controlled study that investigated the effects of a 16-week intake of CGAs on cognitive performance in humans. Comparison between CGA and placebo groups indicated the potential beneficial effects of CGAs on some cognitive functions. 

In the cognitive assessment using Cognitrax, practice effects (defined as improvement in performance due to repeated exposure to test materials and procedures [35,36]), were observed in most of the scores, especially between the 0 W and 8 W scores. Therefore, we performed statistical analysis with two time points (8 W–16 W), including the baseline score as covariance, to isolate the influence of these effects. 

The primary measures of interest of this study were cognitive domain scores (Table 3). Among these, psychomotor speed was significantly increased in the CGA group compared with the placebo group as well as motor speed. The domain score for psychomotor speed in Cognitrax is defined as “how well a subject perceives, attends, responds to complex visual-perceptual information, and performs motor speed and fine motor coordination” [32,33]. The scores for psychomotor speed is calculated from the FTT, one of the most commonly used tests to detect cognitive decline [32,37], and the SDC, a test for the assessment of attention and speed of information processing [32,38]. In the FTT, the scores in the CGA group were significantly increased compared with those in the placebo group. In the SDC, on the other hand, the CGA group showed a tendency toward lower numbers of errors (main effect of group; *p* = 0.080, time × group interaction; *p* = 0.066), which might reflect the improvement of information processing function that is necessary for better psychomotor performance [39].

Considerable evidence highlights the fact that slowing of psychomotor speed is a typical result of normal aging [40,41], and is related to the declines in other cognitive functions such as verbal fluency [42]. In addition, the slowing of psychomotor speed is a risk factor for falls in older adults [43]. Therefore, maintaining psychomotor speed is critical for the elderly, and CGAs could play a beneficial role in this effort, as we have shown here.

The domain score for executive function was also significantly increased in the CGA group compared with the placebo group. The domain score for executive function is defined as “how well a subject recognizes rules, categories, and manages or navigates rapid decision making” [32,33]. In addition, we found that the domain score for cognitive flexibility was higher in the CGA group, although the difference was not statistically significant (time × group interaction; *p* = 0.087). The domain score for cognitive flexibility reflects “how well a subject is able to adapt to rapidly changing and increasingly complex set of directions and/or to manipulate the information” [32,33]. Both executive function and cognitive flexibility scores were calculated using the SAT. The scores for the SAT significantly increased compared with those of the placebo group, indicating improvement in the function of attention control [32]. Considering these findings, CGA intake may improve an individual’s ability to perform complex tasks efficiently by improving not only motor activity but also cognitive functions such as attention control. These findings are in accordance with the results of our previous pilot study, wherein a 6-month intake of CGAs was found to improve cognitive function, especially in the prefrontal cortex [25]. 

Blood analysis showed that the serum concentrations of TTR and ApoA1 increased due to the CGAs (Table 5). TTR confers neuroprotective properties, and decreased levels of TTR in the cerebrospinal fluid are associated with cognitive impairment [44]. The levels of ApoA1, one of the main constituents of high-density lipoproteins, are reduced in the cerebrospinal fluid of AD patients [45]. Of late, these proteins have been gaining considerable attention because recent cross-sectional and longitudinal cohort studies have shown their potential for use as blood biomarkers of early-stage cognitive decline [46]. Therefore, increased TTR and ApoA1 levels might reflect the improved cognitive functions, as observed in the neuropsychological tests (Table 3 and Table 4).

A previous human study reported that a single ingestion of CGAs did not confer any significant benefits with respect to cognitive function [24]. Considering the fact that the duration of our present study was 16 weeks, a relatively long-term intake of CGAs might be necessary for the manifestation of the cognitive-improving effects, which are different from an acute effect as seen with caffeine [24,31]. The proper dosage may also be important since the previous study adopted a higher dose (540 mg). 

Although the fundamental mechanisms underlying the cognition-improving effects of CGAs remain unelucidated, the various properties of CGAs, such as the ones conferring neuroprotection and stimulating neurogenesis, may be key to the effects observed herein (see Introduction). In addition, CGAs have been shown to counter hypertension and obesity [17,29,47,48], both of which are risk factors for cognitive decline, leading to mild cognitive impairment and AD [8]. A recent study has also shown that CGAs can shorten sleep latency, which is the amount of time taken to transition from wakefulness to sleep [28]. This is significant in improving sleep quality, which is essential in maintaining cognitive function, as metabolic waste products in the brain are cleared during sleep [49]. Considering these findings, it is possible that long-term daily intake of CGAs may prevent cognitive disorders not only via direct neuroprotective action, but also indirectly by improving metabolic syndrome and sleep quality. 

To summarize, we investigated the effects of a 16-week intake of CGAs on cognitive functions in middle-aged and elderly individuals, as assessed using a cognitive test battery. The results indicated that CGAs improve some cognitive functions, including attention as well as motor speed, which may facilitate efficient performance of complex tasks. Blood concentrations of TTR and ApoA1—proteins whose reduced levels are markers of early-stage cognitive decline—increased after the CGA treatment, which might reflect the improved cognitive functions observed in the neuropsychological tests. 

A limitation of the present study is that practice effects might occur in some tests, as observed by the increase in the scores of the placebo groups (Table 3 and Table 4). Although practice effects are common in neuropsychological tests involving healthy individuals, they are undesirable and diminish the differences between the experimental and control groups [35,36]. Another limitation was the study period. The intervention period of this study (16 weeks) may be too short to observe the cognitive-improving effects of CGAs in detail, as cognitive decline progresses over the years. In addition, the number of participants was also limited in this study. Further studies with a higher number of participants over longer timescales are necessary, along with reduced practice effects, to confirm the long-term benefits of CGAs for cognitive function. 

## Figures and Tables

**Table 1:** Cognitive domain scores estimated using Cognitrax.

Cognitive Domains	Score Calculations
Neurocognitive Index (NCI)	Average of Five Domain Scores: Composite Memory, Psychomotor Speed, Reaction Time, Complex Attention, Cognitive Flexibility
Composite Memory	VBM (Correct Hits + Correct Passes) + VIM (Correct Hits + Correct Passes)
Verbal Memory	VBM (Correct Hits + Correct Passes)
Visual Memory	VIM (Correct Hits + Correct Passes)
Psychomotor Speed	SDC (Correct Responses) + FTT (Right Tap Average + Left Tap Average)
Reaction Time	ST (Complex Reaction Time + Stroop Reaction Time)
Complex Attention	ST (Stroop Commission Errors) + SAT (Errors) + CPT (Commission Errors)
Cognitive Flexibility	SAT (Correct Responses − Errors) − ST (Stroop Commission Errors)
Processing Speed	SDC (Correct Responses − Errors)
Executive Function	SAT (Correct Responses − Errors)
Simple Attention	CPT (Correct Responses − Commission Errors)
Motor Speed	FTT (Right Taps Average + Left Taps Average)

**Table 2:** Baseline characteristics of participants.

Group	Placebo	CGA
Number of participants (male)	18 (9)	20 (12)
Age, y	58.5 ± 6.3	59.3 ± 5.0
Body weight, kg	63.1 ± 11.4	60.6 ± 11.5
MMSE score	26.6 ± 1.5	26.7 ± 1.4
RBANS total score	49.0 ± 10.7	50.0 ± 16.2

Values represent mean ± SD.

**Table 3:** The cognitive domain scores calculated in Cognitrax.

		Standardized Scores			ANCOVA (*p*-Values)			LMM (*p*-Values)
		Baseline (0 W)	8 W	16 W	T (8 W–16 W)	G (8 W–16 W)	T × G (8 W–16 W)	T × G (0 W–16 W)
Neurocognitive Index (NCI)	Placebo	82.7 ± 20.5	92.2 ± 23.2	95.7± 8.8	<0.05	0.251	0.539	0.970
	CGA	87.5 ± 23.0	97.6 ± 15.9	102.0 ± 9.0				
Composite Memory	Placebo	86.3 ± 17.2	104.7 ± 13.2	97.6 ± 11.0	0.190	0.327	0.107	0.269
	CGA	84.3 ± 25.4	94.8 ± 24.6	99.5 ± 12.9				
Verbal Memory	Placebo	84.3 ± 13.9	105.2 ± 13.0	98.4 ± 16.4	<0.05	0.356	0.212	0.594
	CGA	81.3 ± 25.1	96.8 ± 24.0	98.1 ± 19.1				
Visual Memory	Placebo	94.2 ± 19.5	102.4 ± 13.3	98.3 ± 13.0	0.770	0.629	0.109	0.254
	CGA	92.8 ± 19.3	95.8 ± 17.8	101.3 ± 8.8				
Psychomotor Speed	Placebo	94.9 ± 21.2	99.3 ± 20.6	95.5 ± 11.9	0.141	0.684	<0.05	<0.05
	CGA	91.9 ± 23.9	92.3 ± 28.7	103.4 ± 13.8 ^#^				
Reaction Time	Placebo	82.8 ± 23.4	90.4 ± 13.5	90.7 ± 10.6	0.708	0.305	0.461	0.273
	CGA	101.3 ± 36.5	99.6 ± 27.7	94.4 ± 11.8				
Processing Speed	Placebo	102.3 ± 14.4	106.2 ± 13.6	110.1 ± 11.4	0.946	0.158	0.938	0.937
	CGA	107.7 ± 13.9	112.6 ± 9.3	116.4 ± 13.1				
Motor Speed	Placebo	91.5 ± 25.7	94.8 ± 21.1	86.8 ± 14.3	0.290	0.838	<0.05	<0.05
	CGA	85.5 ± 25.2	84.6 ± 28.2	94.4 ± 14.8				
Complex Attention	Placebo	81.9 ± 29.4	91.7 ± 29.5	98.1 ± 20.7	<0.05	0.132	0.832	0.914
	CGA	88.3 ± 330	101.6 ± 22.3	108.1 ± 12.6				
Simple Attention	Placebo	91.6 ± 27.8	85.8 ± 31.9	90.9 ± 29.6	0.284	0.242	0.567	0.316
	CGA	84.8 ± 35.4	89.3 ± 30.3	100.7 ± 14.1				
Cognitive Flexibility	Placebo	80.8 ± 23.4	95.9 ± 16.4	96.1 ± 14.4	<0.05	0.641	0.087	0.279
	CGA	91.9 ± 18.4	101.0 ± 16.7	104.9 ± 11.9				
Executive Function	Placebo	81.2 ± 23.1	97.0 ± 16.2	96.6 ± 14.6	<0.05	0.794	<0.05	0.145
	CGA	91.8 ± 18.1	100.6 ± 16.8	104.9 ± 11.1 ^#^				

Values represent mean ± SD. ^#^ *p* < 0.1 vs. placebo by unpaired *t*-test. ANCOVA: analysis of covariance. LMM: a mixed linear model. T: time effect analyzed by ANCOVA. G: group effect analyzed by ANCOVA. T × G: time × group interaction analyzed by ANCOVA or LMM.

**Table 4:** The scores of individual tests in Cognitrax.

		Standardized Scores			ANCOVA (*p*-Values)			LMM (*p*-Values)
		Baseline (0 W)	8 W	16 W	T (8 W–16 W)	G (8 W–16 W)	T × G (8 W–16 W)	T × G (0 W–16 W)
Verbal Memory Test (VBM)—Immediate								
Correct Hits	Placebo	89.6 ± 27.4	100.6 ± 23.6	100.2 ± 16.7	0.181	0.685	0.375	0.545
	CGA	82.6 ± 33.6	94.8 ± 34.2	102.0 ± 15.8				
Correct Passes	Placebo	103.7 ± 8.0	105.3 ± 10.9	104.3 ± 7.0	0.203	0.093	0.639	0.739
	CGA	99.8 ± 16.5	101.5 ± 10.0	97.5 ± 14.3				
Verbal Memory Test (VBM)—Delay								
Correct Hits	Placebo	78.4 ± 20.9	104.6 ± 12.2	95.1 ± 15.7	0.154	0.643	0.590	0.669
	CGA	82.2 ± 24.8	99.9 ± 21.7	96.0 ± 25.8				
Correct Passes	Placebo	102.2 ± 11.1	104.7 ± 7.7	100.7 ± 10.9	0.665	0.244	0.244	0.418
	CGA	99.3 ± 15.6	97.0 ± 16.0	100.4 ± 14.6				
Visual Memory Test (VIM)—Immediate								
Correct Hits	Placebo	83.5 ± 27.0	97.4 ± 18.3	90.2 ± 17.7	0.289	0.591	0.081	0.146
	CGA	77.1 ± 32.1	86.3 ± 32.6	94.8 ± 13.1				
Correct Passes	Placebo	106.9 ± 19.6	106.1 ± 16.5	110.2 ± 10.0	0.636	0.629	0.189	0.374
	CGA	107.0 ± 16.8	108.0 ± 12.8	105.3 ± 11.6				
Visual Memory Test (VIM)—Delay								
Correct Hits	Placebo	85.3 ± 21.9	98.7 ± 19.4	93.3 ± 12.4	0.315	0.976	0.121	0.303
	CGA	85.8 ± 31.5	93.8 ± 21.9	98.1 ± 15.7				
Correct Passes	Placebo	109.0 ± 19.4	102.4 ± 17.2	100.8 ± 17.7	0.919	0.879	0.542	0.825
	CGA	110.4 ± 15.9	102.3 ± 14.8	103.4 ± 14.4				
Finger Tapping Test (FTT)								
Right Taps Average	Placebo	92.3 ± 28.0	97.2 ± 17.8	87.4 ± 12.4	0.100	0.922	<0.05	<0.05
	CGA	87.0 ± 25.7	84.8 ± 31.8	96.0 ± 16.0 ^#^				
Left Taps Average	Placebo	93.8 ± 17.2	92.9 ± 23.2	87.9 ± 18.5	0.403	0.535	0.126	0.054
	CGA	85.0 ± 25.4	86.0 ± 25.2	93.5 ± 13.4				
Symbol Digit Coding (SDC)								
Correct Responses	Placebo	102.8 ± 13.0	106.9 ± 11.4	110.6 ± 9.6	0.655	0.261	0.618	0.922
	CGA	107.7 ± 12.6	112.6 ± 9.2	115.3 ± 12.9				
Errors	Placebo	94.8 ± 23.6	95.3 ± 24.0	93.1 ± 18.0	<0.05	0.080	0.066	0.390
	CGA	98.8 ± 20.4	99.1 ± 17.0	106.5 ± 9.1				
Stroop Test (ST)								
Simple Reaction Time	Placebo	76.8 ± 25.4	88.6 ± 14.7	86.6 ± 19.6	0.667	0.310	0.337	0.642
	CGA	64.9 ± 32.5	76.1 ± 26.9	80.6 ± 25.3				
Complex Reaction Time	Placebo	82.3 ± 21.5	87.1 ± 14.5	89.2 ± 9.4	0.080	0.225	0.860	0.367
	CGA	74.9 ± 26.9	89.6 ± 18.6	92.0 ± 11.2				
Stroop Reaction Time	Placebo	85.8 ± 23.3	94.9 ± 12.0	93.9 ± 11.2	0.245	0.721	0.169	0.251
	CGA	81.4 ± 29.7	91.9 ± 21.4	97.3 ± 11.3				
Stroop Commission Errors	Placebo	94.8 ± 17.5	90.7 ± 19.3	96.2 ± 15.8	0.920	0.105	0.075	0.133
	CGA	99.2 ± 13.9	102.9 ± 8.1	99.3 ± 14.8				
Shifting Attention Test (SAT)								
Correct Responses	Placebo	79.3 ± 20.7	94.6 ± 14.9	94.0 ± 13.9	<0.05	0.779	<0.05	0.165
	CGA	88.3 ± 17.7	97.9 ± 17.2	102.2 ± 12.3 ^#^				
Errors	Placebo	88.8 ± 22.5	101.8 ± 14.9	102.1 ± 14.0	<0.05	0.733	0.074	0.168
	CGA	99.8 ± 16.5	105.2 ± 13.2	108.7 ± 7.9				
Correct Reaction Time	Placebo	91.3 ± 14.4	101.6 ± 13.9	101.9 ± 12.8	0.189	0.530	0.214	0.518
	CGA	94.9 ± 15.2	104.5 ± 16.6	107.8 ± 14.7				
Continuous Performance Test (CPT)								
Correct Responses	Placebo	90.0 ± 27.8	78.8 ± 34.5	87.0 ± 32.2	0.561	0.348	0.960	0.434
	CGA	81.0 ± 36.4	83.7 ± 33.4	92.8 ± 20.8				
Commission Errors	Placebo	91.6 ± 28.9	92.6 ± 28.5	96.3 ± 22.2	0.561	0.348	0.960	0.867
	CGA	94.8 ± 27.4	97.2 ± 23.0	103.5 ± 9.4				
Reaction Time	Placebo	78.8 ± 17.4	84.8 ± 19.0	84.8 ± 8.4	<0.05	0.401	0.607	0.965
	CGA	78.4 ± 18.4	86.3 ± 11.7	85.9 ± 9.1				

Values represent mean ± SD. ^#^ *p* < 0.1 vs. placebo by unpaired *t*-test. ANCOVA: analysis of covariance. LMM: a mixed linear model. T: time effect analyzed by ANCOVA. G: group effect analyzed by ANCOVA. T × G: time × group interaction analyzed by ANCOVA or LMM.

**Table 5:** Blood concentrations of Apo A1 and TTR.

		Baseline (0 W)	16 W	∆ Values
Apo A1, mg/dL	Placebo	161.1 ± 29.0	155.2 ± 29.6	−5.9 ± 15.1
	CGA	161.9 ± 27.6	165.9 ± 31.7	4.0 ± 17.4 ^#^
TTR, mg/dL	Placebo	25.9 ± 4.5	24.7 ± 3.8	−1.2 ± 2.5
	CGA	25.7 ± 4.7	26.5 ± 5.1	0.7 ± 2.5 *

Values represent mean ± SD. * *p* < 0.05, ^#^ *p* < 0.1 vs. placebo by unpaired *t*-test.
